# Supplementary material for: Complex small-world regulatory networks emerge from the 3D organisation of the human genome
Source: Nat Commun. 2021 Oct 1;12:5756. doi: 10.1038/s41467-021-25875-y (PMC8486811; doi:10.1038/s41467-021-25875-y)
Supplement: Supplementary file 1 — Supplementary Information [file 41467_2021_25875_MOESM1_ESM.pdf]

Complex small-world regulatory networks emerge from the 3D organisation of the human genome: Supplementary Information

C. A. Brackley *et al.*

Supplementary Figures

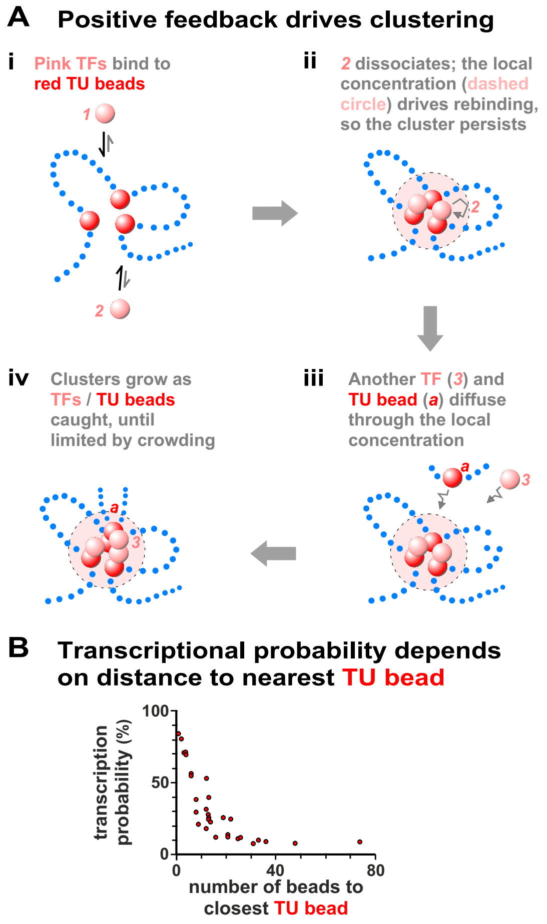

Supplementary Figure 1: **Clusters form spontaneously, and nearby TU beads tend to be transcribed more often.** **A.** Clustering is driven by positive feedback (the "bridging-induced attraction") [1, 2]. (i) Multivalent TFs *1* and *2* (pink) bind reversibly to red TU beads; each blue dot represents many non-binding beads. No other attractive forces between TFs or between TUs are specified. (ii) The two TFs bound; each stabilizes a loop. The local concentration of red TUs in the dashed volume has now increased, so if pink TF *2* dissociates it is likely to rebind to the same cluster (grey arrow); therefore, the cluster is likely to persist. (iii) The high local concentration also drives cluster growth. Here, the cluster will catch red TU *a* and pink TF *3*, as they diffuse through this local region. (iv) Cluster growth continues due to this positive feedback until limited by the entropic costs of crowding together ever more loops. **B.** Scatter plot where each point represents a TU bead, and the horizontal axis gives the distance along the chain in beads to the nearest neighbouring TU. A strong anticorrelation with transcriptional activity is evident. The positive feedback described in (A) ensures that the closer a TU is to another TU, the higher the probability it will cluster and be transcriptionally active.

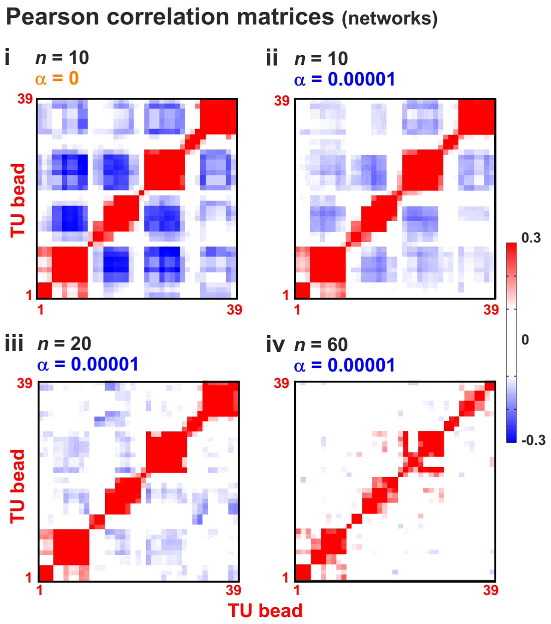

Supplementary Figure 2: **Pearson correlation matrices.** Pearson correlation (covariance) matrices for simulations in Fig. 3 in the main text. These are used to construct regulatory networks.

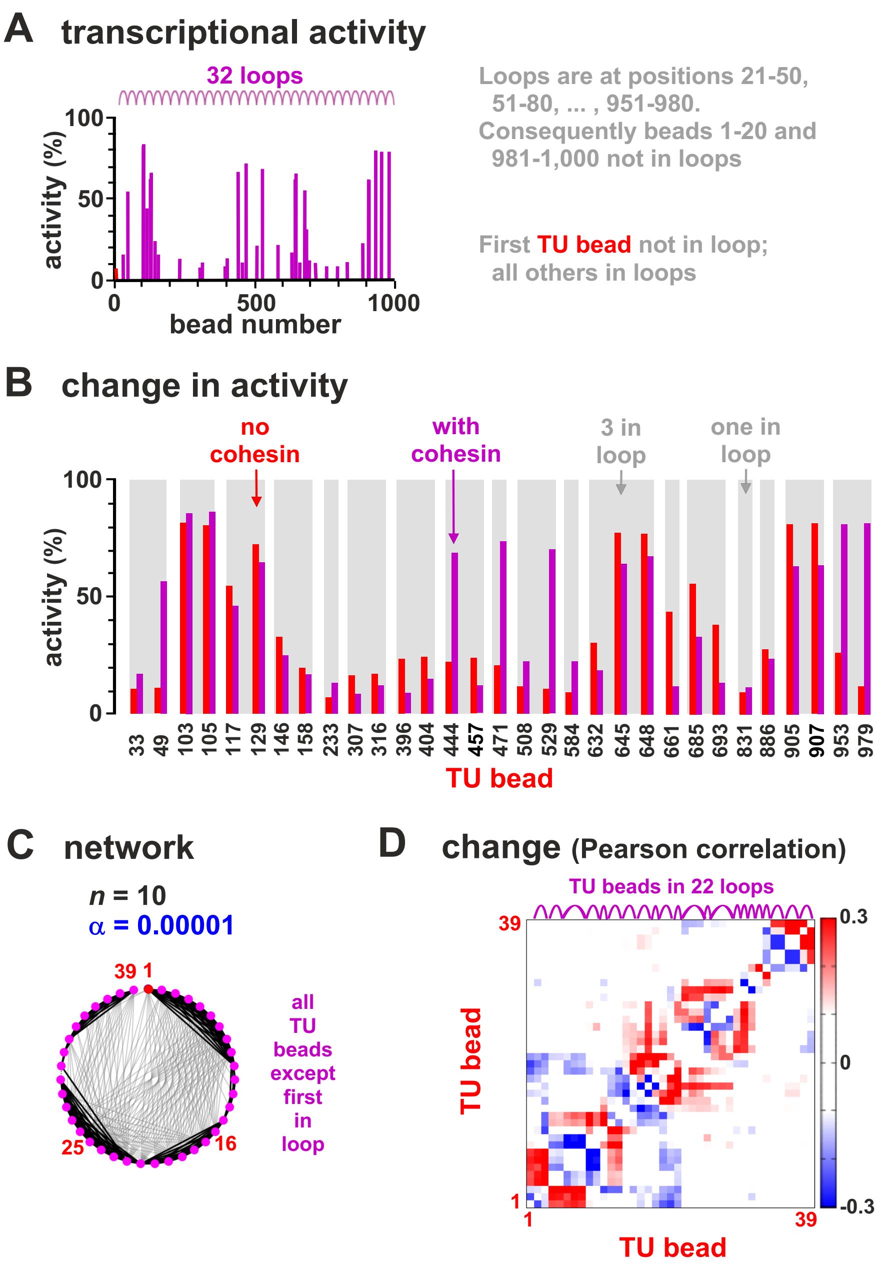

Supplementary Figure 3: **Transcriptional activity in a chain with closely-packed loops.** Results of two sets of simulations ($\geq$ 800 simulations/condition) are compared; one set as Fig. 1 in the main text, in the other the chain contains 32 consecutive and closely-packed permanent loops of size 30 σ. **A.** Average transcriptional activity for each TU in the looped set (magenta bars indicate values for TUs in loops, and magenta arcs loop positions). **B.** Comparison between expression in wild-type and looped configuration for 31 TUs with significantly different values in the two sets ($p \simeq 0.003$; Students t-test). **C.** Regulatory network inferred from the matrix of Pearson correlations between expression of TUs (as Fig. 3A in the main text). **D.** Change in Pearson correlation between TUs due to introduction of loops.

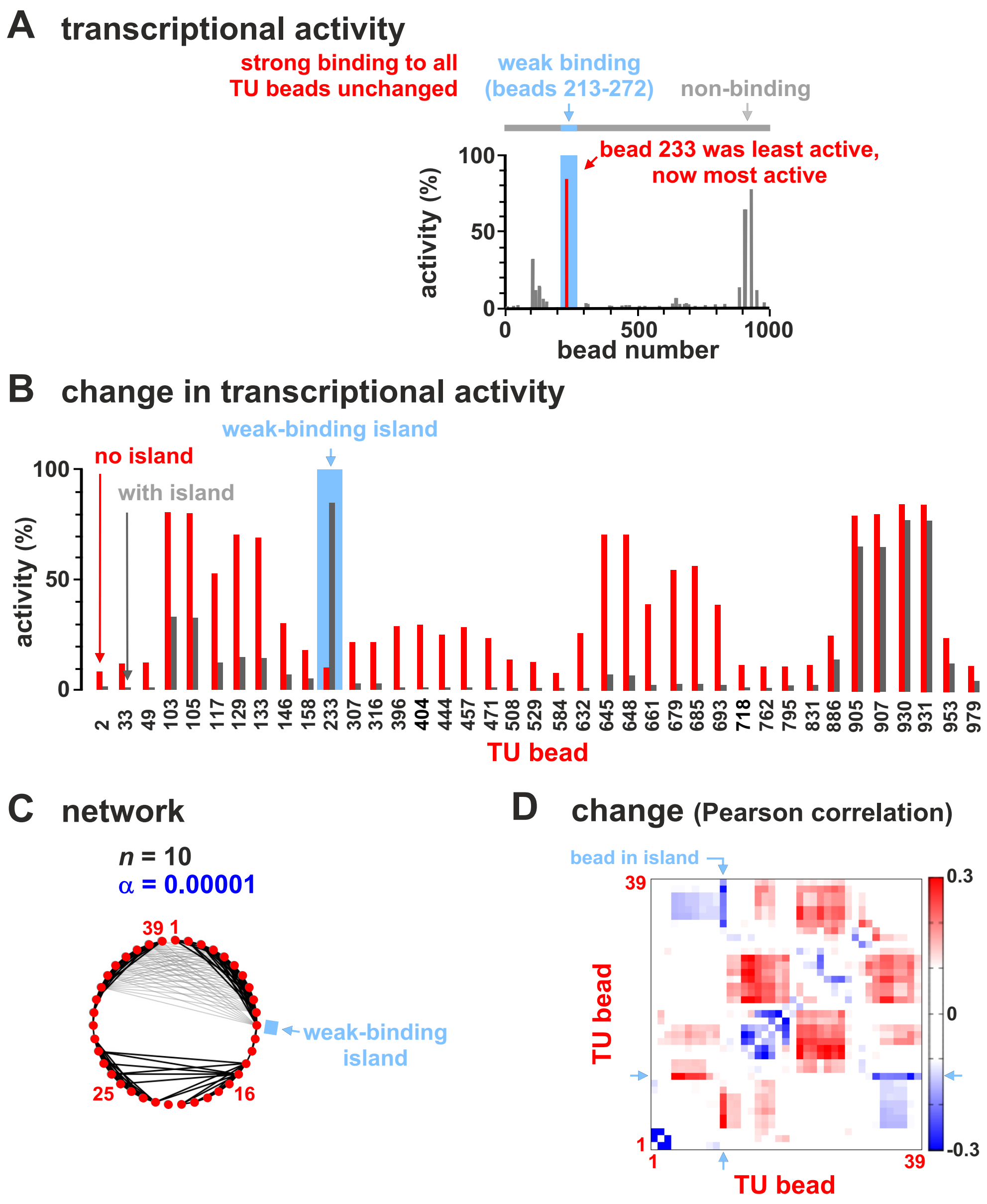

Supplementary Figure 4: **Transcriptional activity in a heterochromatic chain with an euchromatic island.** Results from two sets of simulations (800 simulations/condition) are compared; one set as Fig. 1 in the main text, in the other all non-TU beads are heterochromatic apart from those between bead 213 and 272 inclusive (to represent a euchromatic island). **A.** Average transcriptional activity for each TU bead. Grey bars: values for TUs in heterochromatin. **B.** Comparison of average transcriptional activity with respect to the wild-type for all 39 TUs; these all have significantly-different values in the two sets ($p \simeq 0.003$; Students t-test). **C.** Regulatory network inferred from the matrix of Pearson correlations between transcriptional activities of TUs (as Fig. 3A in the main text). **D.** Change in Pearson correlation between TUs with respect to the wild-type.

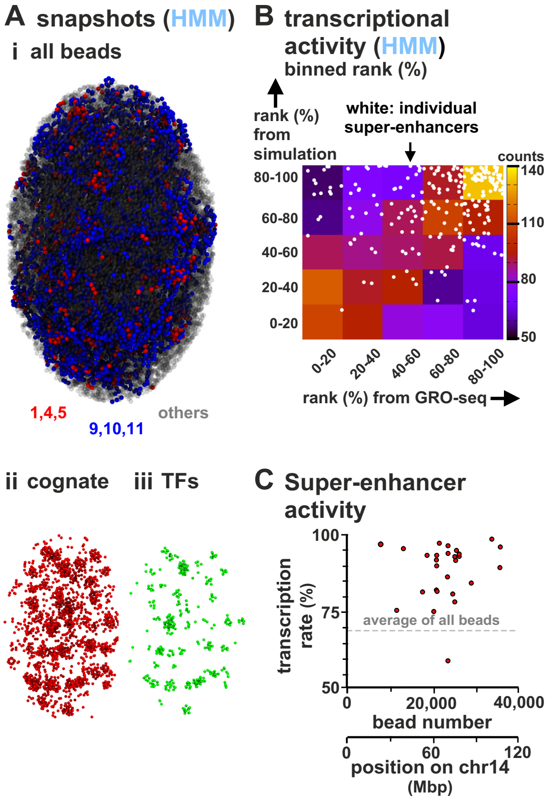

Supplementary Figure 5: **Comparison of transcriptional activities of human chromosome** 14 **in HUVECs determined using simulations (HMM model) and GRO-seq. A. Snapshot.** (i) All beads on the chain (TFs not shown, numbers refer to state numbers in HMM tracks in [3] used to color beads). (ii, iii) TU beads and TFs corresponding to the configuration in (i). **B.** Comparison of transcriptional activities of red TUs in simulations and GRO-seq (ranked from $0-100\%$ and binned in quintiles). A scatter plot of unbinned ranks of beads corresponding to SEs are superimposed (white circles). **C.** Transcription rate of SEs. For a given SE, the rate is the average of all TUs in the SE region. We find 26 of 27 SEs have a higher-than-average expression/transcriptional activity. Data correspond to 100 simulations.

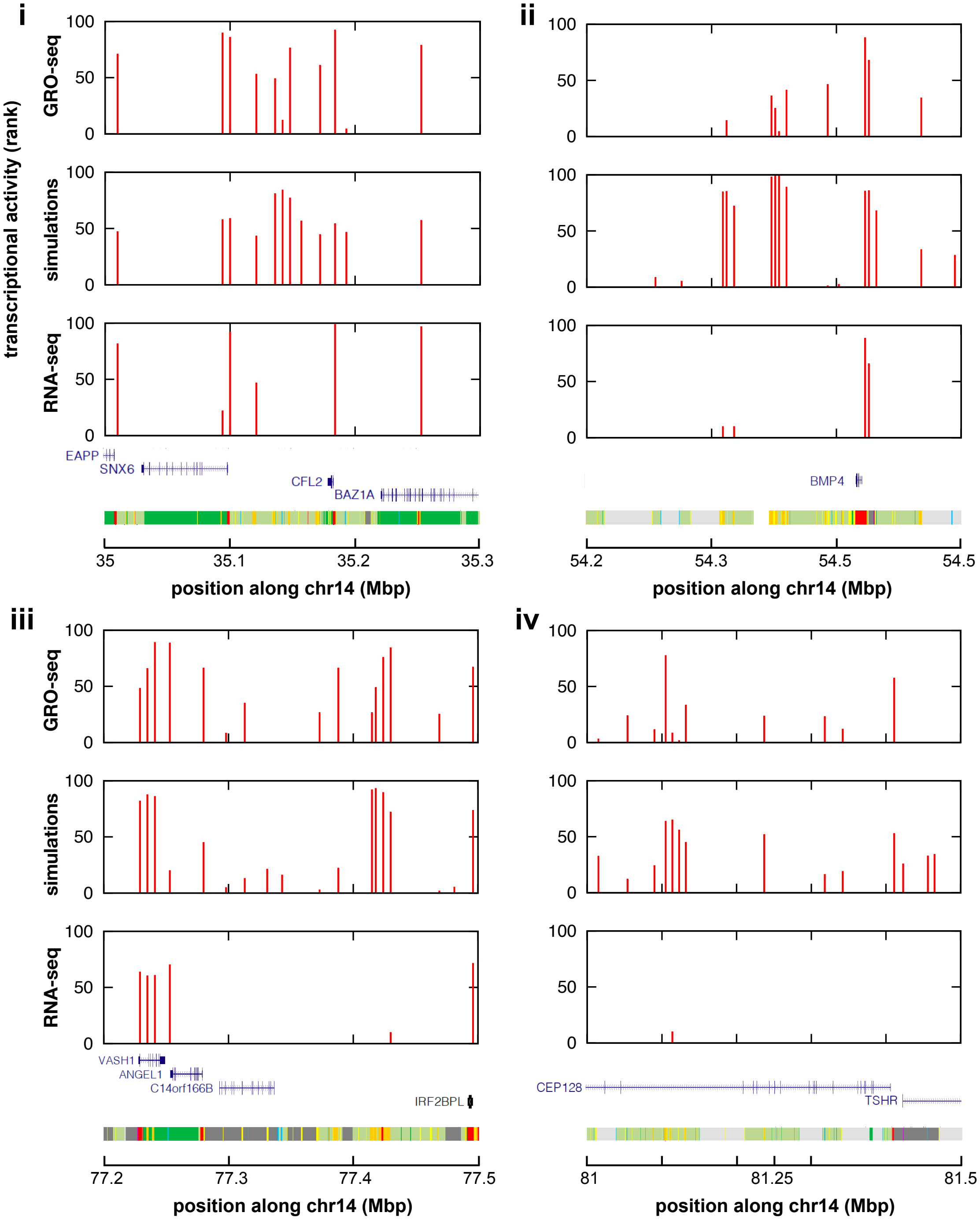

Supplementary Figure 6: **Comparison between transcriptional activity patterns in GRO-seq, simulations and RNA-seq**. **(i-iv).** The three tracks on each of the panels correspond to GRO-seq (top track), simulations (DHS model, middle track) and poly(A)$^+$ RNA-seq (bottom track) for 4 regions of chromosome 14, in HUVECs. To ensure the signals on the y axis have the same scale, we plot the rank of transcriptional activity of DHS beads. The rank is computed over all DHS beads in chromosome 14; for GRO-seq and RNA-seq, we remove data with no signal when computing ranks. The two tracks at the bottom of each panel correspond to the UCSC gene track (without non-coding genes and splice variants), and to the chromatin HMM track [3] for HUVECs. The RNA-seq dataset used was GEO: GSM2072428 [4]. The legend for the colours in chromatin HMM track is as follows [3]: bright red = active promoter; light red = promoter flanking; purple = inactive promoter orange = strong enhancer; yellow = weak enhancer; blue = CTCF/insulator; dark green = transcription; light green = low activity; gray = polycomb repressed; light gray = heterochromatin.

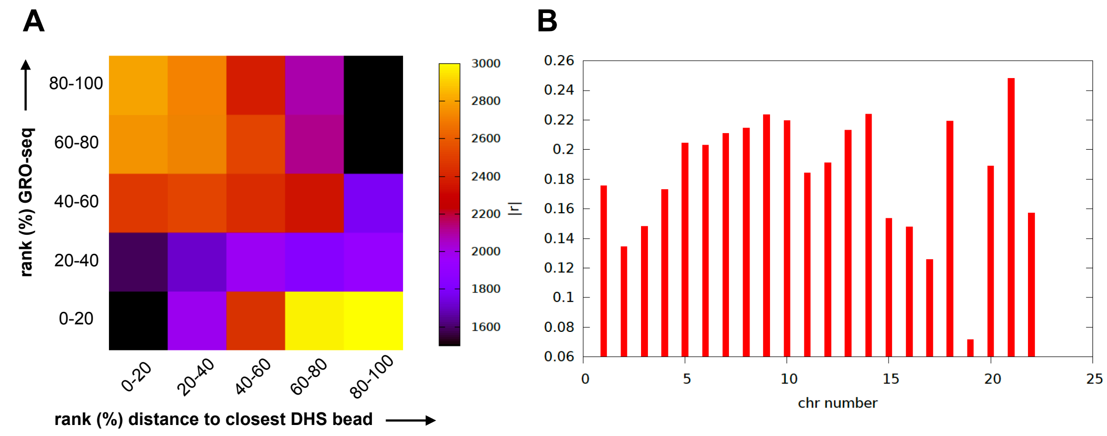

Supplementary Figure 7: **GRO-seq data inversely correlate with distance to nearest DHS peak, and the correlation varies genome-wide. A.** Correlation between the distance to the nearest DHS peak and the GRO-seq signal at DHS peaks genome-wide (ranked from $0-100\%$, then binned in quintiles and shown as a heat map). The heat map shows there is a negative correlation between the two quantities (range in the colour map reduced to highlight the pattern), analogously to the anti-correlation found in our simulations (see Fig. S1) and main text. **B.** The plot shows the absolute value of the Spearman correlation, $|r|$, between GRO-seq and the distance to the nearest DHS peak, for each chromosomes. The negative correlation is typically highly statistically significant: $p < 10^{-12}$ for all chromosomes except 16, 17, 19, 22, for which $p \simeq 5 \times 10^{-12}$, 5×10^{-12}, ~ 0.0007, 4×10^{-8} respectively. To compute the correlations, we coarse grain the GRO-seq data and DHS peaks into 1 kbp segments.

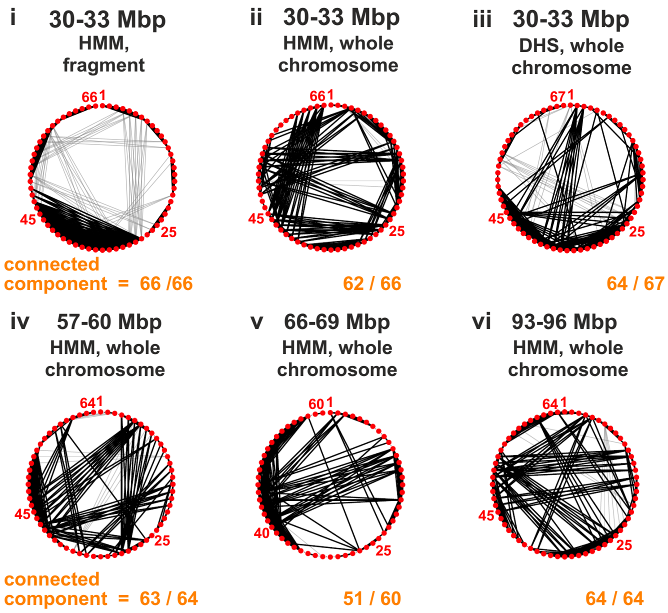

Supplementary Figure 8: **Regulatory networks in chains representing fragments of human chromosome 14 in HUVECs are highly connected.** Networks are constructed and presented as for Fig. 3 in the main text; they involve Pearson correlations between transcriptional activities of all TUs/red beads. To facilitate comparison with results in Fig. 3 in the main text, four fragments of the chain representing chromosome 14 are selected that have the same length as the 3 Mbp chain (Fig. 3 in the main text), and roughly the same density of TUs. (i) Dilute conditions (volume fraction $< 0.1\%$). Simulations (800 runs) involve a short 3 Mbp chain in a cube under the dilute conditions used for the 3 Mbp fragment simulations (as Fig. 3 in the main text), but with beads coloured using the HMM model (as Fig. 7 in the main text). Consequently, the statistical certainty associated with this panel is inevitably higher than in the other panels as ≥ 8-fold more runs are involved. (ii-vi) Confined conditions (volume fraction $\sim 14\%$). Simulations (100 runs) are conducted using the DHS and HMM models, and a string representing the whole chromosome in an ellipsoid (as Fig. 7 in the main text, and Fig. S5). Networks in all panels are highly connected. Comparison of panels (i) and (ii) – which allow comparison of the effects of confinement – points to confinement increasing the number of distant positive correlations (as might be expected). Comparison of panels (ii) and (iii) which allow comparison of the HMM and DHS models highlights the different patterns of marks contained within the two models. Comparison of panels (ii)-(vi) shows different segments of the chromosome have highly-connected components.

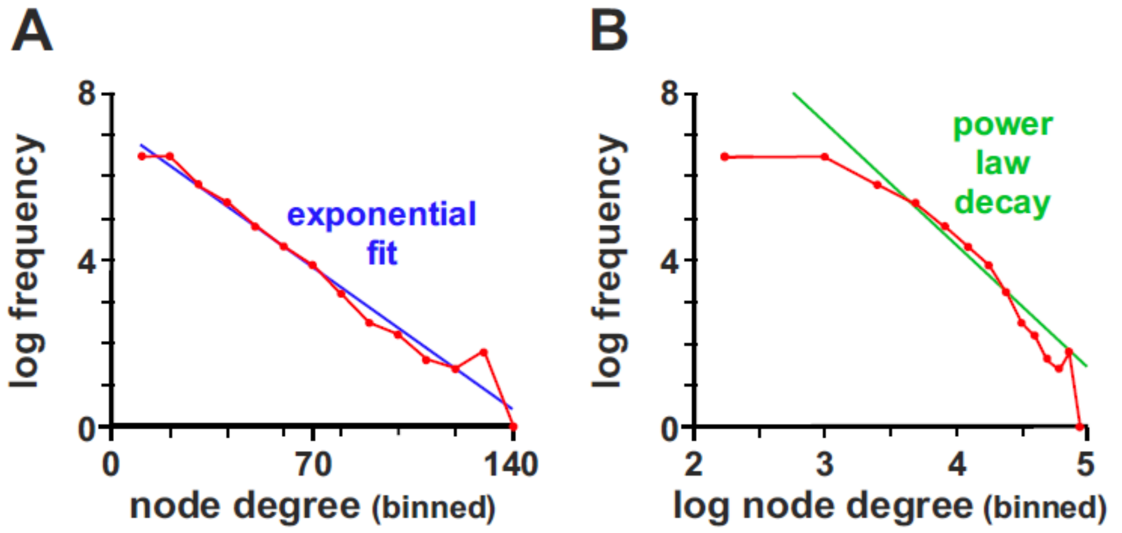

Supplementary Figure 9: **Node-degree distribution for transcriptional networks emerging from simulations (HMM model, HSA14, HUVECs).** An edge between two nodes is drawn when the Pearson correlation between corresponding TU beads is more than 0.35 in absolute value. **A.** Log-linear plot. The blue solid line is a linear fit, corresponding to an exponentially decreasing node-degree distribution. **B.** Log-log plot. The green line is a linear fit for intermediate node degree, corresponding to a power-law decay in the node-degree distribution (expected for a scale-free network). The power-law fit is poorer than the exponential one in (A).

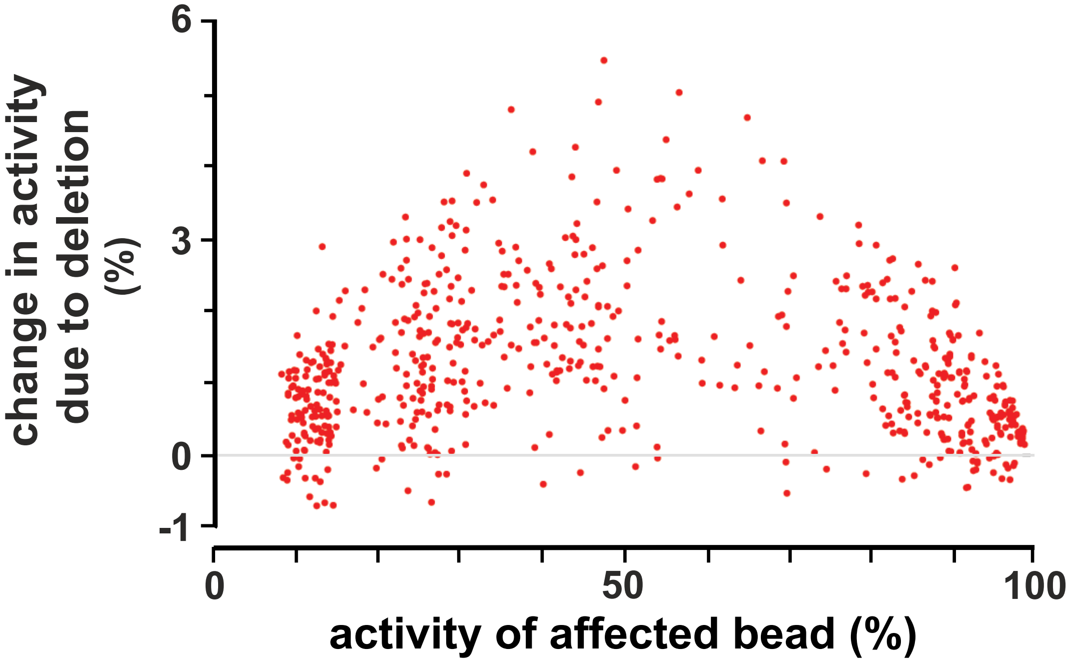

Supplementary Figure 10: **Change in transcriptional activity versus TU activity for DiGeorge deletion.** For each TU, we compute both the change in transcriptional activity in the chromosome 22 deletion studied in Fig. 8 in the main text, as well as the average transcriptional activity in the wild-type and deletion. This scatter plot shows that the change is larger for TUs with intermediate activity.

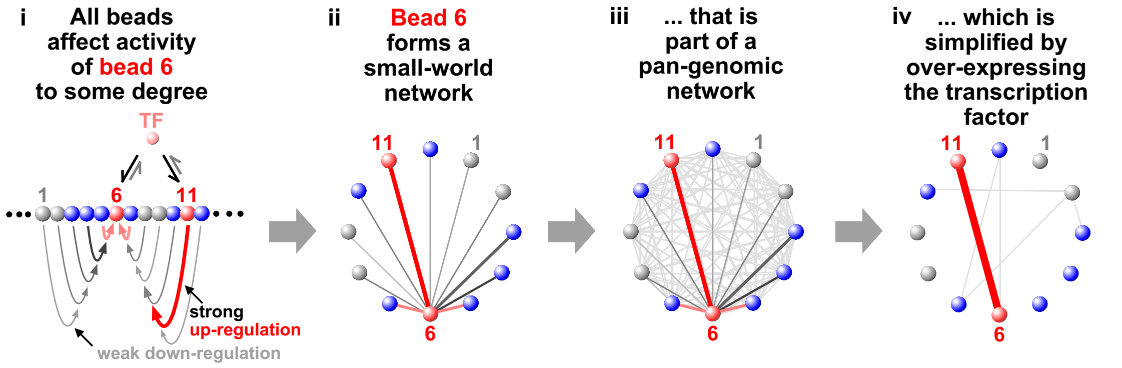

Supplementary Figure 11: **A pan-genomic model for transcriptional organisation and gene regulation.** (i) A region of the human genome is depicted with 12 segments (grey – heterochromatin, blue – open chromatin, red – transcription unit). TFs (transcription factor – polymerizing complexes) are present at non-saturating concentrations; they bind tightly to TUs *6* and *11*, weakly to blue segments, and not to heterochromatin. All other beads in the chain influence transcription of bead *6*. They can be considered as eQTLs acting on bead *6*, with eQTL strength being indicated by curved arrows (red – up-regulation, grey – down-regulation; increasing width/colour indicates increasing strength). (ii) The regulatory network centred just on bead 6. Straight lines indicate interaction strength (colour code as i). (iii) The regulatory network of all 12 beads. Every bead has some effect on the transcription of every other bead, which is consistent with GWAS results. (iv) Increasing TF copy-number (as in reprogramming experiments [5]) simplifies both the network of bead *6*, and the complete network. Consequently, over-expressing the TF over-rides the regulatory network mediated by 3D structure, and allows the coexisting trans-acting network envisioned by the omnigenic model to dominate.

Supplementary Note 1: Artificial chromatin fragment simulations

For the 3 Mbp chromatin fragment simulations, chains are made of 1000 beads (so that each bead corresponds to 3 kbp). For the simulations shown, TUs were placed at beads 2, 33, 49, 103, 105, 117, 129, 133, 146, 158, 233, 307, 316, 396, 404, 444, 457, 471, 508, 529, 584, 632, 645, 648, 661, 679, 685, 693, 718, 762, 795, 831, 886, 905, 907, 930, 931, 953, 979. Different simulations with different TU bead locations (randomly scattered along the fibre with average distance between 10 and 40) give qualitatively similar results, equivalent to the ones shown in the main text. The linear TU density in our simulations (between on average one bead in 20 and one in 40, or between 0.025 and 0.1) encompasses the range relevant to human chromatin. For instance, our whole chromosome simulations of HSA14 and HSA22 (see next Section) correspond to an average TU density (equivalently, density of 3 kbp bead corresponding to DHS peaks) of ~ 0.06 and ~ 0.04 respectively. The ratio between the number of active TFs, n, over the number of TUs, N, which we consider vary between 0.26 and 2.06, with most results presented for the former case. For this case, TFs are not enough to saturate TUs, which is the realistic case (see next section).

To start simulations, the chromatin fibre is initialised as a random walk, and proteins are initialised at random position within the simulation box – a cube of size 100σ, which means the system is dilute. To avoid bead overlap, we initially perform a small number of steps with a soft potential between beads, and a harmonic bond between neighbouring beads. We then equilibrate the system for 10^4 Brownian times with repulsive (WCA) interactions between any pair of beads. Finally we study the evolution of the system for 10^5 Brownian times (10^6 Brownian times for Fig. 2 in the main text) once the attractive Lennard-Jones interactions between chromatin beads and TFs are included in the potential.

The main parameter determining qualitative behaviour is the ratio n/N (number of active TFs to number of TUs). The magnitude of the attractive interaction between TFs and TUs and switching rate do not affect results qualitatively, provided the bridging-induced attraction drives clustering (i.e., we always get variable TU activity, stochasticity at the single-cell level and pangenomic transcriptional regulation dependent on 3D structure). The magnitude of low-affinity interactions, the interaction range r_c, and the persistence length of the chromatin fibre all affect transcriptional patterns and interactions quantitatively, but do not modify qualitative trends.

Supplementary Note 2: Transcriptional difference

To determine the difference between two sets of simulations in Figure 4 in the main text, which we refer to as states (typically corresponding to two different parameter sets), we define a transcriptional difference as follows. If there are N TU beads in the polymer, let us call x_i the expression (transcriptional activity, in %) of the

i-th TU bead in the first state, and x'_i its expression in the second state. The transcriptional difference between the two states is then

$$d_T = \sqrt{\sum_{i=1}^{N} (x'_i - x_i)^2}, \tag{S1}$$

which is the Euclidean distance between the two points $\{x_1, \cdots, x_N\}$ and $\{x'_1, \cdots, x'_N\}$ in their N-dimensional space.

Supplementary Note 3: Whole chromosome simulations

To simulate HSA 14 and HSA22 in a diploid G0 HUVEC cell, the system is confined into an ellipsoidal territory with aspect ratio chosen according to typical experimental values [6, 7] (semiaxes were 22.24σ:34.24σ:41.80σ, or 0.67 μm:1.03 μm:1.25 μm, for HSA14; 17.39σ:26.77σ:32.68σ, or 0.52 μm:0.80 μm:0.98 μm, for HSA22). Confinement is enforced by modifying the source code in LAMMPS to describe an ellipsoidal indenter. This introduces a soft force towards the centre of the ellipsoid, only if beads exit the confining ellipsoid. The magnitude of the confining force felt by a bead at position vector (x, y, z) is

$$f = K_g \delta g^2 \tag{S2}$$

for $\delta g > 0$, where

$$\delta g/a = \sqrt{x^2/a^2 + y^2/b^2 + z^2/c^2} - 1 \tag{S3}$$

where a, b and c are the semiaxes (along x, y and z), and where we assume for simplicity that the centre of the ellipsoid is in $(x, y, z) = (0, 0, 0)$. The constant K_g measures the stength of the confining force and is set to $10 k_B T/\sigma^2$. As a detail we note that the confinement is modelled via an effective force, rather than via an effective potential. Polymer size (in beads) for the simulated chromosomes are 35784 for HSA14, 17102 for wild-type HSA22, and 16250 for HSA22 with diGeorge deletion (again, each bead corresponds to 3 kbp).

There are about $50000 - 60000$ Pol II molecules in a diploid cell [8], corresponding to a ~ 2 nM concentration for a nucleoplasmic volume of ~ 520 μm^3 (a spherical nucleus of diameter 10 μm). FRAP shows that about a quarter of the Pol II molecules exchanges rapidly [9] and are likely to be active at any time. To reflect this, we consider 1700 and 813 TFs for HSA14 and HSA22 simulations respectively (in HUVEC cells), with $k_{\rm off} = 4k_{\rm on} = 0.001$ $\tau_{\rm B}$, so that 20% of these are active on average at any time. As the number of TUs in our DHS model is respectively 2226 (HSA14) and 687 (HSA22), the value of n/N (ratio between average number of TFs active at any time and number of TUs) is ~ 0.15 (HSA14) and ~ 0.24 (HSA22), similar to values considered in the toy model (see previous section).

Our DHS model is described in the main text. Our HMM model is based on the HMM Broad chromatin track [3], which classifies chromatin segments into 15 different states using a hidden Markov model (HMM) applied to a range of

epigenetic marks (Suppl. Fig. S5). Here, states 1, 4, 5 – marking strong promoters and enhancers – are colored red (strong binding), while states 9, 10, and 11 – marking other transcribed regions – are blue (weak binding).

To start simulations, the chromatin fibre is again initialised as a random walk, with soft potential used to avoid overlap. The resulting self-avoiding chain is confined into the ellipsoidal territory quickly, analogous to what is done to create fractal globule structures. We additionally equilibrated the system for 10^4 Brownian times with repulsive (WCA) interactions between any pair of beads, before turning on the protein-chromatin interactions (after which again we collect data for 10^5 Brownian times). While this protocol is not sufficient to reach equilibrium for this long polymer [10], the fractal globule-like structure we choose for the initialisation corresponds to the one normally associated with interphase chromatin [11]. Additionally, as transcriptional activity depends on cluster formation which is predominantly associated with local chromatin folding – a much faster process than global folding [12] – we do not expect the initial condition to affect our conclusions and results. For instance, the chromosome fragment simulations start from an equilibrium configuration (see above), and the main results are qualitatively in line with those of the whole-chromosome simulations.

Supplementary Note 4: Quantification of small-world-ness

To quantify small-world-ness of a network, we use one of the approaches described in [13]. Thus, we create a number (typically 100) of randomised networks where the same number of edges as in the original network are randomly assigned to a a node pair. For both the original and the randomised network, we then compute: (i) the average over nodes of the clustering coefficient [14], which measures how close the neighbours of a node are to forming a clique, or a fully connected graph, and (ii) the average shortest path between a node pair. We call the average clustering coefficients γ and γ_r, for the original and a randomised network respectively, and the average shortest path L and L_r, for the original and a randomised network respectively. The small-world-ness can then be defined as $s = \frac{\gamma}{\langle \gamma_r \rangle} \frac{\langle L_r \rangle}{L}$, where $\langle \cdot \rangle$ denote averaging over many randomised networks. A value of $s > 1$ signals a small-world network. It should be noted that s can only computed with this definition for connected networks, for which L and L_r are well defined.

For the neworks in Fig. 3 of the main text, $s \simeq 1.90$ for $n = 10$ and $s \simeq 2.94$ for $n = 20$ (other networks are not connected). For chromosome 14, $s \sim 1.13$ for the DHS network (with threshold on absolute value of correlation 0.2 to draw an edge between a pair of nodes), and $s \sim 1.27$ for the HMM network (with threshold 0.2). For chromosome 22, $s \sim 6.63$ for the DHS networks and $s \sim 2.29$ for the HMM network (both with threshold 0.2). Therefore, all the networks we can characterise are small-world; it would be interesting to further probe changes in s with n/N and with chromosome region, and to understand the reasons for any significant pattern in the changes in s genome-wide.

Supplementary Note 5: Genomic datasets used

In this Section we detail the genomic datasets used to prepare inputs and compare

outputs in simulations of chromosome HSA 14 and 22 (Figs. 7, 8 in the main text and Suppl. Fig. S5).

Inputs to color beads in the DHS model are based on DNase-hypersensitivity (DHS) and H3K27ac peaks for HUVECs and hESCs, from ENCODE. Beads containing a peak in H3K27ac but not in DHS are colored as low-affinity (blue) beads, and those containing a peak in DHS are colored as high-affinity (red) beads. Beads which did not contain any peak (either DHS or H3K27ac) are colored as non-binding (gray). To color beads in the HMM model (HUVECs only), we use the chromatin states in [3] as detailed in Supplementary Note 3. Our simulations are based on the GRCh37-hg19 genome assembly for inputs.

To compare the predicted transcriptional activity of chromosome 14 outputted by our simulations with experiments, we use GRO-seq data. For HUVECs, we use the datasets GEO: GSM2486801, GSM2486802, GSM2486803 [15]; for hESCs, we use GEO: GSM1579367, GSM1579368 [16]. Super-enhancer regions considered here are those identified in [17], and available in the dbSUPER database, which includes super-enhancers for human and mouse cells.

Supplementary References

[1] Chris A. Brackley, Stephen Taylor, Argyris Papantonis, Peter R. Cook, and Davide Marenduzzo. Nonspecific bridging-induced attraction drives clustering of dna-binding proteins and genome organization. *Proc. Natl. Acad. Sci. USA*, 110:E3605–E3611, 2013.

[2] Chris A. Brackley, James Johnson, Steven Kelly, Peter R. Cook, and Davide Marenduzzo. Simulated binding of transcription factors to active and inactive regions folds human chromosomes into loops, rosettes and topological domains. *Nucleic Acids Res.*, 44:3503–3512, 2016.

[3] Jason Ernst, Pouya Kheradpour, Tarjei S Mikkelsen, Noam Shoresh, Lucas D Ward, Charles B Epstein, Xiaolan Zhang, Li Wang, Robbyn Issner, Michael Coyne, et al. Mapping and analysis of chromatin state dynamics in nine human cell types. *Nature*, 473:43, 2011.

[4] ENCODE Project Consortium. An integrated encyclopedia of dna elements in the human genome. *Nature*, 489(7414):57–74, 2012.

[5] Kazutoshi Takahashi, Koji Tanabe, Mari Ohnuki, Megumi Narita, Tomoko Ichisaka, Kiichiro Tomoda, and Shinya Yamanaka. Induction of pluripotent stem cells from adult human fibroblasts by defined factors. *Cell*, 131(5):861–872, 2007.

[6] Yejun Wang, Mallika Nagarajan, Caroline Uhler, and GV Shivashankar. Orientation and repositioning of chromosomes correlate with cell geometry–dependent gene expression. *Mol. Biol. Cell*, 28:1997–2009, 2017.

[7] P. R. Cook and D. Marenduzzo. Transcription-driven genome organization: a model for chromosome structure and the regulation of gene expression tested through simulations. *Nucleic Acids Res.*, 46(19):9895–9906, 2018.

[8] P. R. Cook. *Principles of nuclear structure and function.* Wiley New York, 2001.

[9] Hiroshi Kimura, Kimihiko Sugaya, and Peter R Cook. The transcription cycle of rna polymerase ii in living cells. *J. Cell Biol.*, 159:777–782, 2002.

[10] Guang Shi, Lei Liu, Changbong Hyeon, and Dave Thirumalai. Interphase human chromosome exhibits out of equilibrium glassy dynamics. *Nat. Comm.*, 9(1):1–13, 2018.

[11] Leonid A Mirny. The fractal globule as a model of chromatin architecture in the cell. *Chromosome Res.*, 19(1):37–51, 2011.

[12] Davide Michieletto, Davide Marenduzzo, and Ajazul H Wani. Chromosome-wide simulations uncover folding pathway and 3d organization of interphase chromosomes. *arXiv preprint arXiv:1604.03041*, 2016.

[13] Mark D Humphries and Kevin Gurney. Network small-world-ness: a quantitative method for determining canonical network equivalence. *PLoS ONE*, 3(4):e0002051, 2008.

[14] Marcus Kaiser. Mean clustering coefficients: the role of isolated nodes and leafs on clustering measures for small-world networks. *New J. Phys.*, 10(8):083042, 2008.

[15] Henri Niskanen, Irina Tuszynskax, Rafal Zaborowski, Merja Heinäniemi, Seppo Ylä-Herttuala, Bartek Wilczynski, and Minna U Kaikkonen. Endothelial cell differentiation is encompassed by changes in long range interactions between inactive chromatin regions. *Nucleic Acids Res.*, 46(4):1724–1740, 2017.

[16] Conchi Estarás, Chris Benner, and Katherine A Jones. Smads and yap compete to control elongation of β-catenin: Lef-1-recruited rnapii during hesc differentiation. *Mol. Cell*, 58(5):780–793, 2015.

[17] Aziz Khan and Xuegong Zhang. dbsuper: a database of super-enhancers in mouse and human genome. *Nucleic Acids Res.*, 44(D1):D164–D171, 2015.
